# Supplementary material for: Non-enzymatic roles of human RAD51 at stalled replication forks
Source: Nat Commun. 2019 Sep 27;10:4410. doi: 10.1038/s41467-019-12297-0 (PMC6764946; doi:10.1038/s41467-019-12297-0)
Supplement: Supplementary file 1 — Supplementary Information [file 41467_2019_12297_MOESM1_ESM.pdf]

Supplementary Information

NON-ENZYMATIC ROLES OF HUMAN RAD51 AT STALLED REPLICATION FORKS

Mason et al.

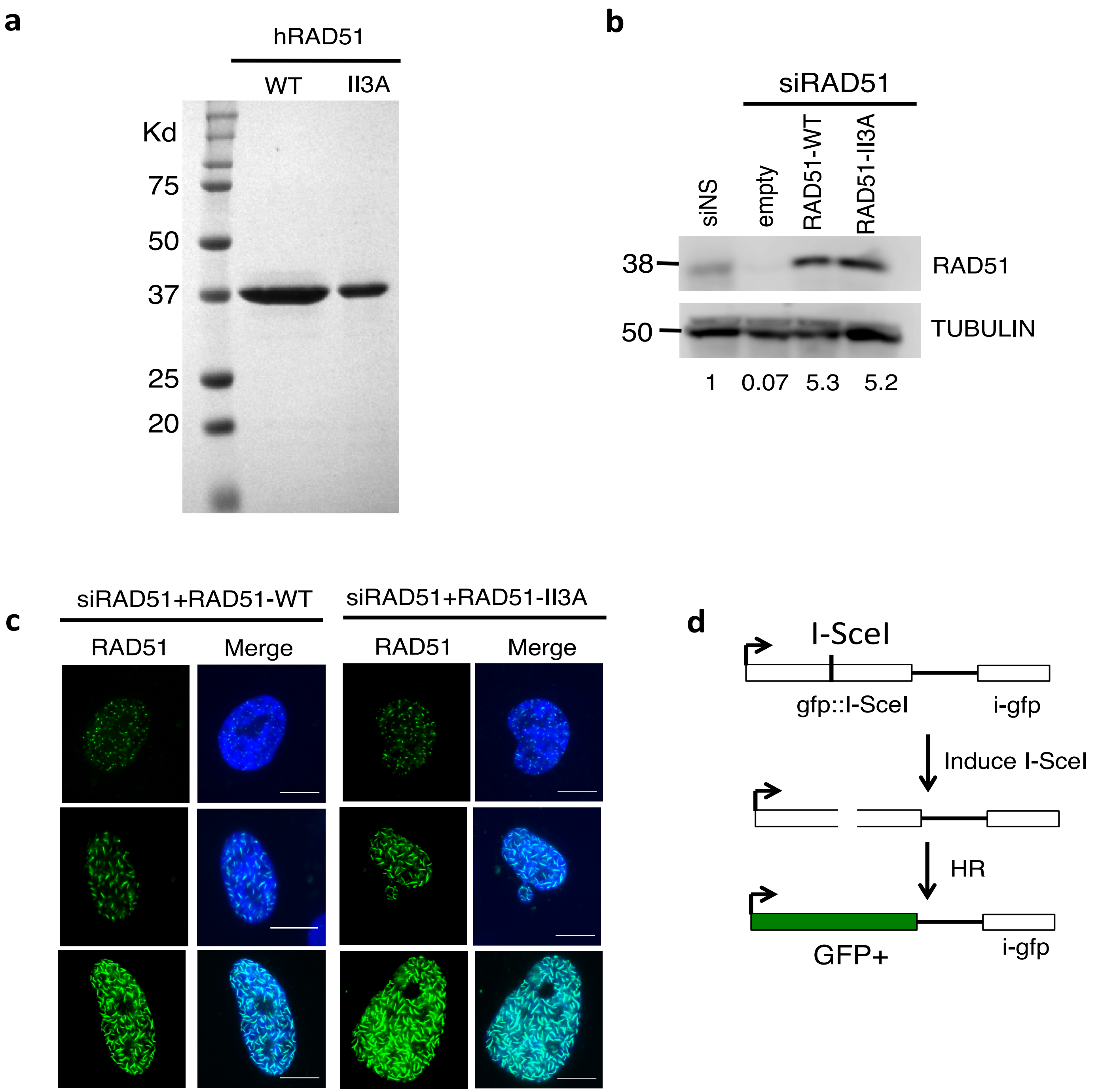

Supplementary Figure 1. Cells over-expressing hsRAD51-WT or hsRAD51-II3A. (a) Purified hRad51-WT (25 µg) and hRad51-II3A mutant (10 µg) were analyzed on a 12% SDS-PAGE and the proteins were stained with Coomassie brilliant blue R250. (b) Western blot depicting levels of RAD51 after indicated treatments. TUBULIN was used as a loading control. The level of RAD51 protein levels (normalized to TUBULIN) relative to the siNS control are indicated below the blot. (c) Representative images of RAD51 fibers (non-damage associated complexes) in a small subpopulation of cells transfected with hsRAD51-WT or hsRAD51-II3A expression constructs. Green- RAD51. Blue-DNA. Scale bar = 10 µm. (d) Depiction of the DR-GFP assay to measure homologous recombination in cells. The GFP coding sequence is disrupted by an I-SceI nuclease site and contains an internal GFP fragment downstream. Repair of the I-SceI induced DSB by HR restores the GFP coding sequence. Thus, HR efficiency is measured by determining the percentage of cells expressing GFP. Source data are provided as a source data file.

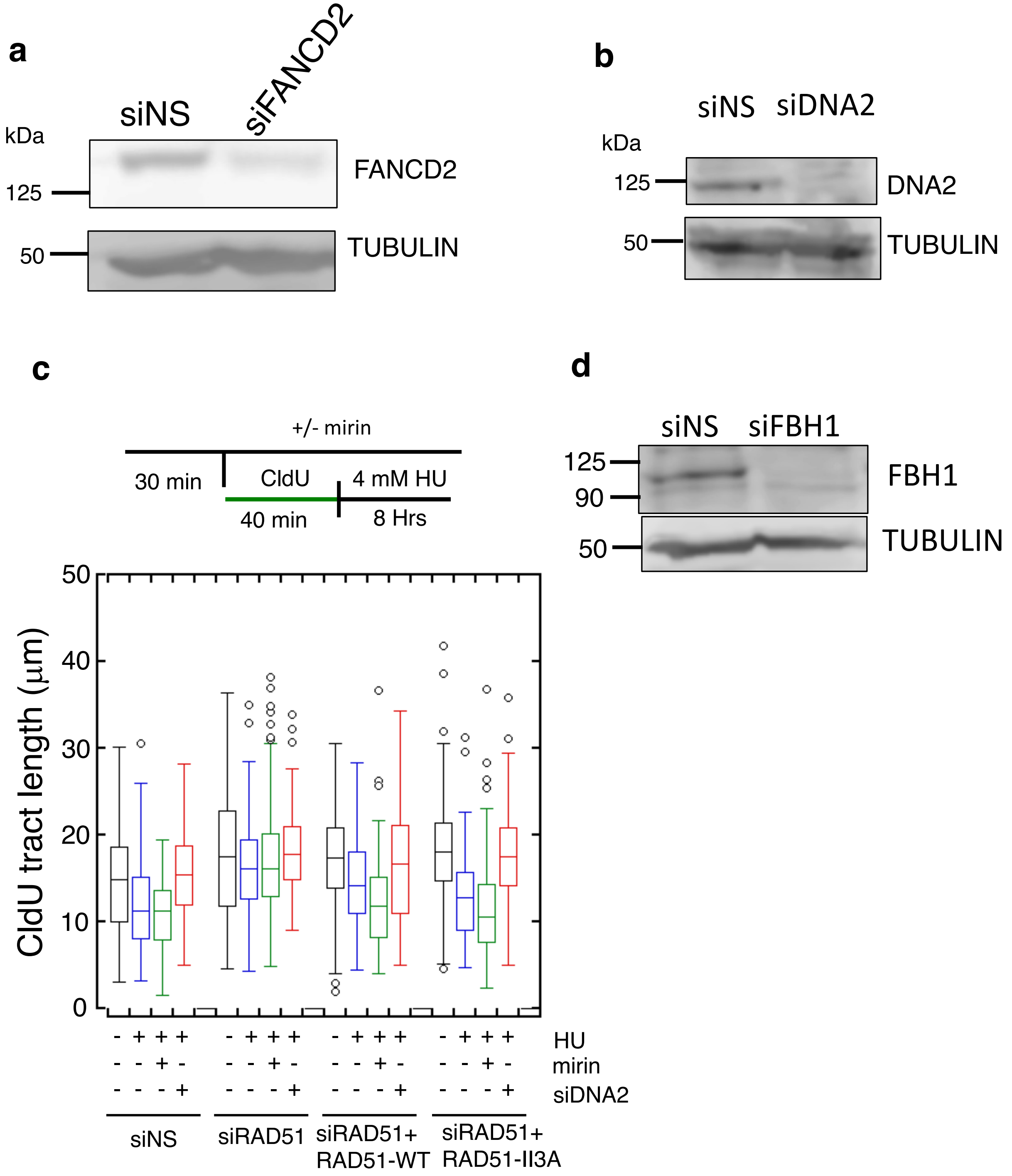

Supplementary Figure 2. **Degradation of replication tracts in RAD51-II3A expressing cells.** (a) Western depicting level of FANCD2 after indicated treatments. TUBULIN was used as a loading control. (b) Western depicting level of DNA2 after the indicated treatments. TUBULIN was used as a loading control. (c) Box plot represents the CldU tract length after the indicated treatment. Lines represent the median for each set of measurements. Boxes represent 25th and 75th quartiles with Tukey whiskers (see methods for details). Data represent at least 100 replication tracts from two independent experiments. Values are presented as mean±STD. Statistical significance was determined using Mann-Whitney Test. Schematic of experimental design is depicted above the graphs. (d) Western depicting level of FBH1 after the indicated treatments. TUBULIN was used as a loading control. Source data are provided as a source data file.

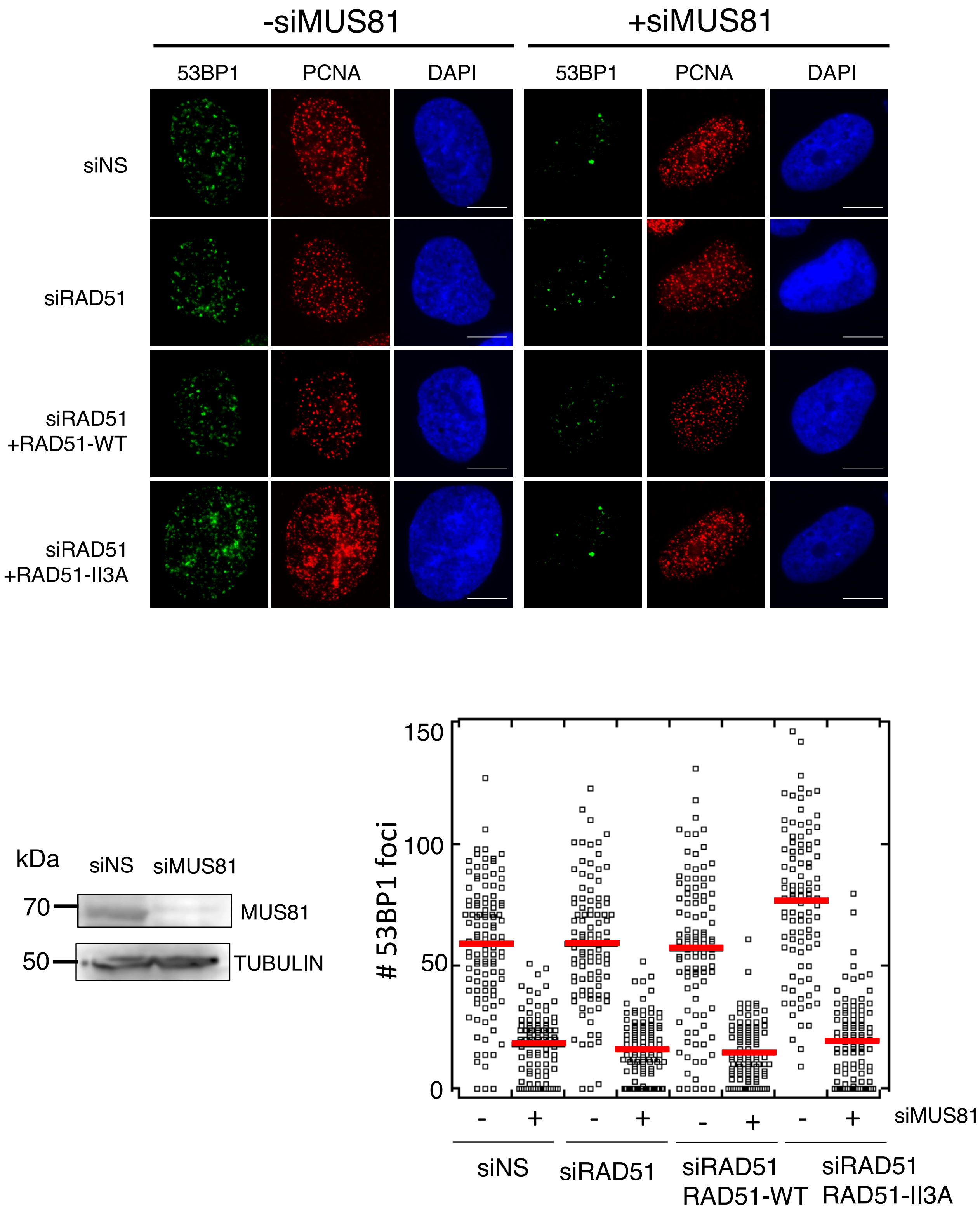

Supplementary Figure 3. 53BP1 accumulates in cells after 24 hours of HU treatment. Representative images depicting 53BP1 foci (green) in PCNA (red) positive cells after treatment with 4mM HU for 24 hours. DNA is stained in blue. Scale bar= 10 μm. Data is combined data consisting of at least 100 nuclei from two independent experiments. Red line represents the mean. Values are presented as mean±STD. Statistical significance was determined using the Mann-Whitney test. Source data are provided as a source data file.

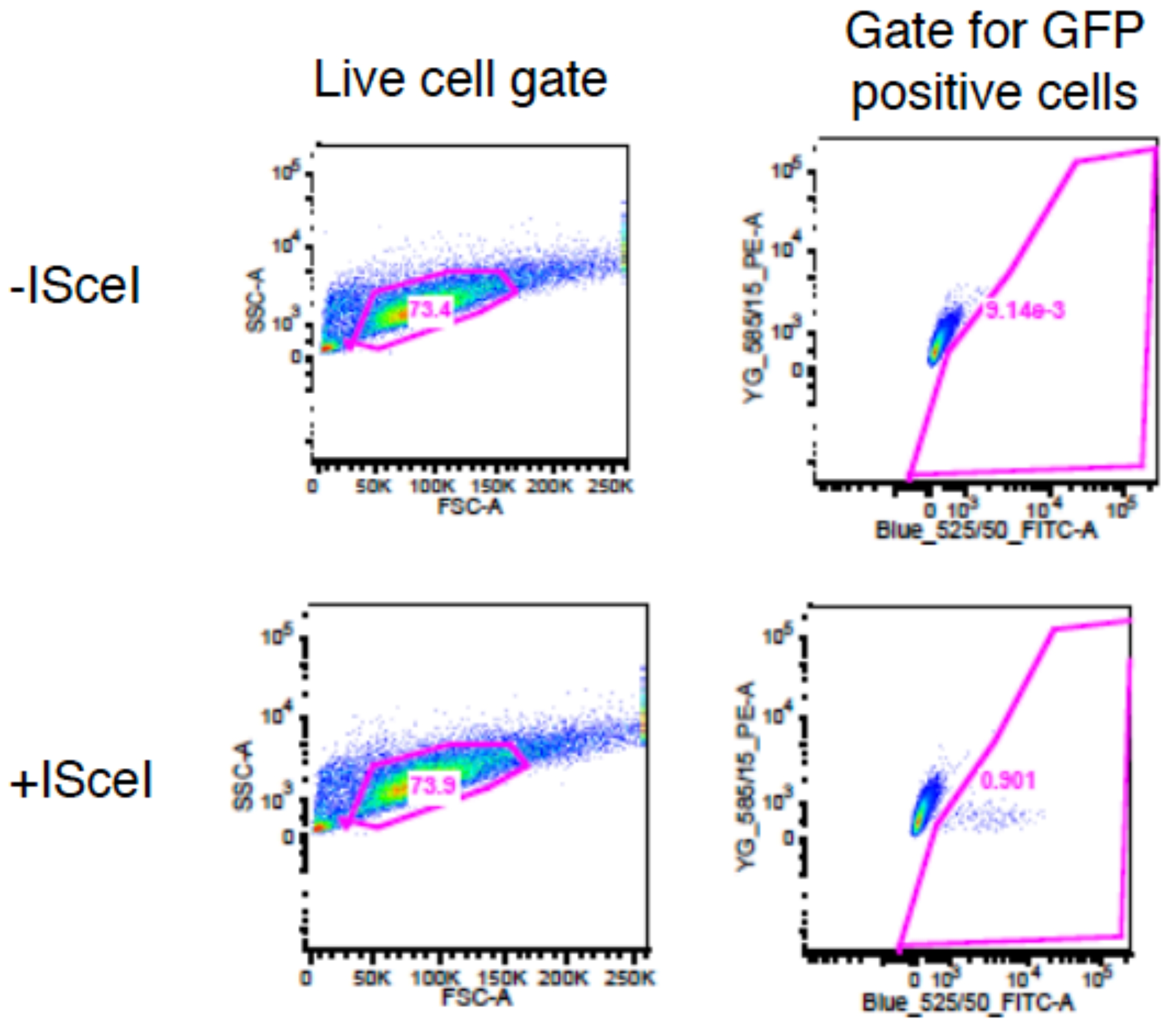

Supplementary Figure 4. Gating strategy for DR-GFP assay. Left panels: U2OS cells without expression of the nuclease I-SceI were used to gate for live cells based on FSC and SSC. The gate was applied to all remaining samples. The gate is shown in pink with the percentage of cells within the gate noted. Right panel: The GFP positive gate was drawn on the negative control population (top) and applied to the remaining samples (bottom). The percent GFP positive cells is denoted and the gate is shown in pink
