# Supplementary material for: Non-enzymatic roles of human RAD51 at stalled replication forks
Source: Nat Commun. 2019 Sep 27;10:4410. doi: 10.1038/s41467-019-12297-0 (PMC6764946; doi:10.1038/s41467-019-12297-0)
Supplement: Supplementary file 2 — Reporting Summary [file 41467_2019_12297_MOESM2_ESM.pdf]

nature research

Corresponding author(s): Jennifer Mason, Douglas K Bishop

Last updated by author(s): Aug 21, 2019

Reporting Summary

Nature Research wishes to improve the reproducibility of the work that we publish. This form provides structure for consistency and transparency in reporting. For further information on Nature Research policies, see Authors & Referees and the Editorial Policy Checklist.

Statistics

For all statistical analyses, confirm that the following items are present in the figure legend, table legend, main text, or Methods section.

n/a	Confirmed	
☐	☒	The exact sample size (*n*) for each experimental group/condition, given as a discrete number and unit of measurement
☐	☒	A statement on whether measurements were taken from distinct samples or whether the same sample was measured repeatedly
☐	☒	The statistical test(s) used AND whether they are one- or two-sided<br>*Only common tests should be described solely by name; describe more complex techniques in the Methods section.*
☒	☐	A description of all covariates tested
☒	☐	A description of any assumptions or corrections, such as tests of normality and adjustment for multiple comparisons
☐	☒	A full description of the statistical parameters including central tendency (e.g. means) or other basic estimates (e.g. regression coefficient) AND variation (e.g. standard deviation) or associated estimates of uncertainty (e.g. confidence intervals)
☐	☒	For null hypothesis testing, the test statistic (e.g. *F*, *t*, *r*) with confidence intervals, effect sizes, degrees of freedom and *P* value noted<br>*Give P values as exact values whenever suitable.*
☒	☐	For Bayesian analysis, information on the choice of priors and Markov chain Monte Carlo settings
☒	☐	For hierarchical and complex designs, identification of the appropriate level for tests and full reporting of outcomes
☒	☐	Estimates of effect sizes (e.g. Cohen's *d*, Pearson's *r*), indicating how they were calculated

Our web collection on statistics for biologists contains articles on many of the points above.

Software and code

Policy information about availability of computer code

Data collection	NA
Data analysis	Image J, DNA Prism, Kaleidagraph, FloJo software

For manuscripts utilizing custom algorithms or software that are central to the research but not yet described in published literature, software must be made available to editors/reviewers. We strongly encourage code deposition in a community repository (e.g. GitHub). See the Nature Research guidelines for submitting code & software for further information.

Data

Policy information about availability of data

All manuscripts must include a data availability statement. This statement should provide the following information, where applicable:
- Accession codes, unique identifiers, or web links for publicly available datasets
- A list of figures that have associated raw data
- A description of any restrictions on data availability

Source data are provided as a source data file. All data supporting the findings of this study are available from the corresponding authors upon request.

Field-specific reporting

Please select the one below that is the best fit for your research. If you are not sure, read the appropriate sections before making your selection.

☒ Life sciences ☐ Behavioural & social sciences ☐ Ecological, evolutionary & environmental sciences

Life sciences study design

All studies must disclose on these points even when the disclosure is negative.

Sample size	No statistical methods were used to determine sample size. Sample sizes were selected based on previous work with similar assyas or based on sample sized employed by others in the field.
Data exclusions	No data were excluded
Replication	All experiments were repeated independently 2 or 3 times. In the case of the DR-GFP assay, the samples expressing RAD51 were also plated in duplicate to account for transfection variability. All independent experiments gave similar restuls.
Randomization	The study involved treated cells with expressing WT or a mutant version of RAD51 and comparing these samples to a RAD51 knockdown control. Because we were comparing specific genotypes to each other in cultured cells, no randomization was required. For any experiments involving DNA protein localization, images of 50-100 random nuclei were acquired based on DNA staining only.
Blinding	For all replication fiber assays, the investigators were blind to the sample identity during acquisition and analysis.

Reporting for specific materials, systems and methods

We require information from authors about some types of materials, experimental systems and methods used in many studies. Here, indicate whether each material, system or method listed is relevant to your study. If you are not sure if a list item applies to your research, read the appropriate section before selecting a response.

Materials & experimental systems

n/a	Involved in the study	
☐	☒	Antibodies
☐	☒	Eukaryotic cell lines
☒	☐	Palaeontology
☒	☐	Animals and other organisms
☒	☐	Human research participants
☒	☐	Clinical data

Methods

n/a	Involved in the study	
☒	☐	ChIP-seq
☐	☒	Flow cytometry
☒	☐	MRI-based neuroimaging

Antibodies

Antibodies used	RAD51 is a rabbit polyclonal antibody against purified human RAD51 (1:1000, Pacific Immunology). 53BP1 (1:1000, NB100-304) was from Novus Biologicals and PCNA (1:1000, IG7) was from Abnova, Anti-DNA2 (1:500; ab96488), Anti-MUS81 (1:1000, ab14387), and anti-FBH1 (1:100, ab58881) were from Abcam.
Validation	RAD51,DNA2, MUS81, FBH1 were all validated using siRNA knockdown (Western blots are in supplementary). Novus biologicals validated the 53BP1 antibodies using a 53BP1 knockout as stated on the website.

Eukaryotic cell lines

Policy information about cell lines

Cell line source(s)	U2OS cells with a stably integrated copy of the DR-GFP plasmid was generated in the lab of Dr. Maria Jasin
Authentication	Cell line was validated by short tandem repeat profiling at the Genetic Resources Core facility
Mycoplasma contamination	All cell lines tested negative for mycoplasma contamination with regular checks by DAPI staining of nuclei
Commonly misidentified lines (See ICLAC register)	No commonly misidentified cell lines were used in this study

Flow Cytometry

Plots

Confirm that:

☒ The axis labels state the marker and fluorochrome used (e.g. CD4-FITC).

☒ The axis scales are clearly visible. Include numbers along axes only for bottom left plot of group (a 'group' is an analysis of identical markers).

☒ All plots are contour plots with outliers or pseudocolor plots.

☒ A numerical value for number of cells or percentage (with statistics) is provided.

Methodology

Sample preparation	Cells were collected, pelleted, and resuspended in 1X PBS
Instrument	BD Biosciences LSR II
Software	Samples were collected on the BS Biosciences LSR II using the FACsDiva Software. Analysis of images were done using
Cell population abundance	n/a
Gating strategy	Followed routine gating strategy for live cells. A negative control (GFP negative) were used to generate the gate for GFP positive cells

☒ Tick this box to confirm that a figure exemplifying the gating strategy is provided in the Supplementary Information.
